# Sexually aggressive behavior triggered by parasitic infection – how parasites can influence our personality

**DOI:** 10.3389/fpsyt.2025.1555024

**Published:** 2025-05-27

**Authors:** Marco Goczol

**Affiliations:** ^1^ Department of Forensic Medicine, Medical Faculty, Leipzig University, Leipzig, Germany; ^2^ Department of National Security, Faculty of Information Sciences, University of Library Studies and Information Technologies, Sofia, Bulgaria

**Keywords:** personality change, parasitic infection, sexual aggression, Toxoplasma gondii, neuropsychiatric, psychological behavior

## Abstract

Parasitic infections are not only a health problem, but also a psychological and behavioral one. Research shows that certain parasites can influence the personality traits and behavior of infected individuals. Toxoplasma gondii, a well-known parasite, is suspected of increasing the risk of sexually aggressive behavior. This paper examines the links between parasitic infections and personality changes and analyzes the mechanisms by which parasites can affect the nervous system and associated behaviors. The aim is to raise awareness of the psychological and behavioral effects of parasitic infections and to stimulate future research in this area.

## 1 How parasites affect the way people think and act

Parasitic infections are a common but often underestimated phenomenon worldwide, which can have not only physical but also psychological and behavioral effects. While most people associate parasitic infections primarily with physical symptoms, research is increasingly showing that certain parasites can have profound effects on the central nervous system and thus on the personality and behavior of those infected (1). A well-known example is *Toxoplasma gondii*, a parasite that is often transmitted through contact with cats or contaminated food. Studies suggest that infection with *T. gondii* can affect the behavior and personality of humans and other animals, and in some cases is even associated with an increase in risky or aggressive behavior (2, 3). To make matters worse, this parasite is probably one of the most common in host species other than humans and is the most common parasite in humans. It is thought that 60-80% of older people are infected with this parasite (4). This aspect is particularly interesting with regard to sexually aggressive behavior, as there is evidence that parasitic infections can cause profound behavioral changes that not only influence individual experience but also have potential social consequences (5). Once infected with the parasite, the latent, inactive phase of infection lasts for many years in people with healthy immune systems, and the parasite remains viable (6). Such findings raise important questions: To what extent can parasites manipulate human behavior? What are the biological mechanisms underlying these changes? And what are the implications for our understanding of human behavior and the prevention of behavioral disorders?

Several vector-borne infections, such as *Toxoplasma gondii*, *Trypanosoma brucei* (causative agent of African sleeping sickness), *Borrelia burgdorferi* (causative agent of Lyme disease), *Bartonella henselae* (causative agent of cat scratch disease), *Plasmodium* spp. (malaria pathogen) and *Babesia* spp. (causative agent of babesiosis), are known to cause neuropsychiatric and behavioral symptoms in their hosts (7). Parasites have evolved to manipulate their hosts in ways that increase their own chances of survival and spread. This paper aims to provide an overview of the link between parasitic infections and sexually aggressive behavior and to highlight the mechanisms by which parasites can affect human behavior. Analysis of existing studies will provide a better understanding of how parasites have the potential to alter the personality and behavior of their hosts. Ultimately, the work should help to raise awareness of the psychological and behavioral effects of parasites and contribute to research in this field.

## 2 Background and theoretical framework

*Toxoplasma gondii*, an intracellular parasite, is known to live in the intestines of cats in its sexual reproductive phase but can spread to a wide range of mammals, including humans. Research shows that the parasite can affect the behavior of its hosts, particularly in rodents, by forming cysts in the brain. For example, infected rats show a reduced fear of cats, which is called “fatal attraction” and helps the parasite to return to its main host, the cat (3).

Research on humans has shown that subtle but significant personality changes can also occur in us as a result of infection with *T. gondii*. For example, it was found that infected men and women show different personality changes, such as an increased willingness to take risks and a reduced reaction speed. Such effects are particularly interesting because they not only influence individual behavior but could also have societal implications (8). These behavioral changes can be traced back to the mechanisms by which *T. gondii* affects neurotransmitter balance and neuronal function. Studies suggest that the parasite may act on dopamine balance, which could lead to increased vulnerability in people with mental illnesses such as schizophrenia (2). In addition, there is evidence that other parasites can also manipulate the behavior of their hosts. For example, parasites from the nematode family influence the behavior of their hosts to complete the infection cycle. These manipulation strategies underscore how parasites can use biochemical and genetic mechanisms to adjust host behavior in ways that are beneficial for parasite survival and dispersal (9). This theoretical framework provides the basis for understanding the ways in which parasites might affect human behavior and shows that behavioral changes as a result of parasitic infections are not rare or random occurrences, but rather deeply rooted evolutionary strategies.

### 2.1 Basics of parasitic infections and their interaction with the human organism

Parasitic infections affect a wide range of biological systems and can be caused by a wide range of organisms, including protozoa, worms and arthropods. These parasites often have complex life cycles and specific host organisms in which they can reproduce or develop. A well-known example of such a parasite is *T. gondii*, a protozoan that can live in a variety of warm-blooded animals and is known for its unique interactions with the human organism (10). Parasites often enter the human body through the digestive tract, skin or blood and then systematically spread. Once inside the host, they find ways to evade or manipulate the immune system. For example, *T. gondii* can hijack immune cells such as macrophages to hide within these cells and systematically infect the body, including the central nervous system (11). Once in the brain, the parasite forms cysts in neuronal tissues, where it can remain for a long time without being eliminated by the immune system.

*Naegleria fowleri* is an amoeba-like protozoan that occurs in warm freshwater and in rare cases can cause a serious infection in humans. If the amoebae enter the body through the nose while swimming, they migrate along the olfactory nerve into the brain, where they cause primary amoebic meningoencephalitis (PAM). The disease begins with symptoms such as headache, fever, nausea and vomiting and progresses rapidly to stiff neck, confusion, hallucinations and coma. The infection is usually fatal. Despite the use of various medications, the survival rate is extremely low (4, 12).

Some parasites, such as *Trypanosoma brucei*, the causative agent of African sleeping sickness, can infect the central nervous system, causing neurological symptoms and behavioral changes. Similarly, larvae of the pork tapeworm (*Taenia solium*) can enter the human brain, encapsulate there and damage the surrounding tissue, which can lead to inflammation with neurological symptoms and behavioral changes. There is also increasing evidence that infections could play a role in neurodegenerative processes such as Alzheimer’s or Parkinson’s disease (4, 13, 14).

Bartonella infections, especially those caused by *Bartonella henselae*, the causative agent of cat scratch disease, can cause neuropsychiatric manifestations in addition to the typical physical symptoms. These manifestations are varied and can affect the central nervous system. Possible neuropsychiatric symptoms of Bartonellosis can include sudden restlessness and irritability, panic attacks, treatment-resistant depression and severe psychotic symptoms such as hallucinations or delusions (15).

Piroplasmas, in particular *Babesia* spp. (causative agent of babesiosis) and *Plasmodium* spp. (causative agent of malaria), are protozoa that infect red blood cells and can cause various clinical manifestations. Although the primary symptoms are often hematological and systemic in nature, both infections can also cause neuropsychiatric manifestations. Babesiosis typically manifests with non-specific symptoms such as fever, headache and muscle pain. However, in severe cases, especially in immunocompromised individuals, neurological symptoms similar to those of malaria may also occur. These include confusion or even coma, seizures, paralysis or speech disorders. Malaria is particularly associated with behavioral changes due to psychotic episodes and cognitive impairment (4, 16).

*Wolbachia* is a bacterium that occurs in 40% of arthropod species (arachnids, crustaceans, millipedes and insects). There they manipulate the reproduction of the host and thereby influence the formation of species (17). Transmission to humans takes place via mosquitoes. Mosquitoes suck blood infected with *W.* mircrofilariae, which develop into larvae in the mosquito, which in turn enter the human bloodstream via the proboscis through the bite canal during the next blood meal. After a long incubation period, unspecific symptoms appear, followed by late symptoms such as swelling of the lymph nodes. The consequences can include elephantiasis tropica, enormous swelling of the extremities, the genital area and the mammary glands (4).

### 2.2 Overview of the influence of parasites on the central nervous system and possible effects on behavior

The influence of parasites on the central nervous system and human behavior is increasingly being investigated in scientific research. Parasites such as *T. gondii* are able to alter the normal function of the brain by producing neurotransmitter-like molecules or by influencing signal transduction. For example, it has been shown that *T. gondii* increases dopamine production in host cells. Dopamine is an important neurotransmitter associated with motivation, reward, and certain risky or aggressive behaviors (18).

*T. gondii* infection is associated with various psychological and behavioral changes. Studies show that infected individuals may exhibit increased risk-taking and decreased reaction speed, which is explained by the parasite’s effect on the dopaminergic system (8).

The ability of parasites to influence the behavior of their hosts is therefore not just a side effect of the infection, but often appears to be an evolutionarily advantageous mechanism for the parasites themselves. Altering neurotransmitter production and manipulating neuronal function in the host are central mechanisms by which parasites such as *T. gondii* gain control over host behavior (19).

### 2.3 Mechanisms of behavioral influence

Parasitic infections often influence host behavior in subtle but significant ways. A central mechanism by which parasites such as *T. gondii* and others cause behavioral changes is by affecting neurotransmitter balance in the central nervous system. Studies suggest that *T. gondii* can affect the production of neurotransmitters such as dopamine in host cells, which can have significant behavioral effects (18). A correlation was found between latent infection with *T. gondii* and personality traits such as aggressiveness and impulsivity, a subtle change in the behavior of individuals infected with *T. gondii* was detected (20).

#### 2.3.1 Biological mechanisms

*Toxoplasma gondii* infection leads to cyst formation in the brain, particularly in regions involved in behavior management and emotions, such as the limbic system. These cysts can affect host cell function, with dopamine playing a key role. Dopamine is central to the regulation of reward, motivation, and certain risky or impulsive behaviors (21). Thus, by directly increasing dopamine production in infected cells, *T. gondii* can indirectly influence the host’s risk-taking and behavioral patterns, which can lead to impulsive decisions and an increased tendency to take risks (8). *T. gondii* is associated with mental disorders, suicidal behavior (22) and a tendency to increased risk in road traffic (23).

**Figure 1:**
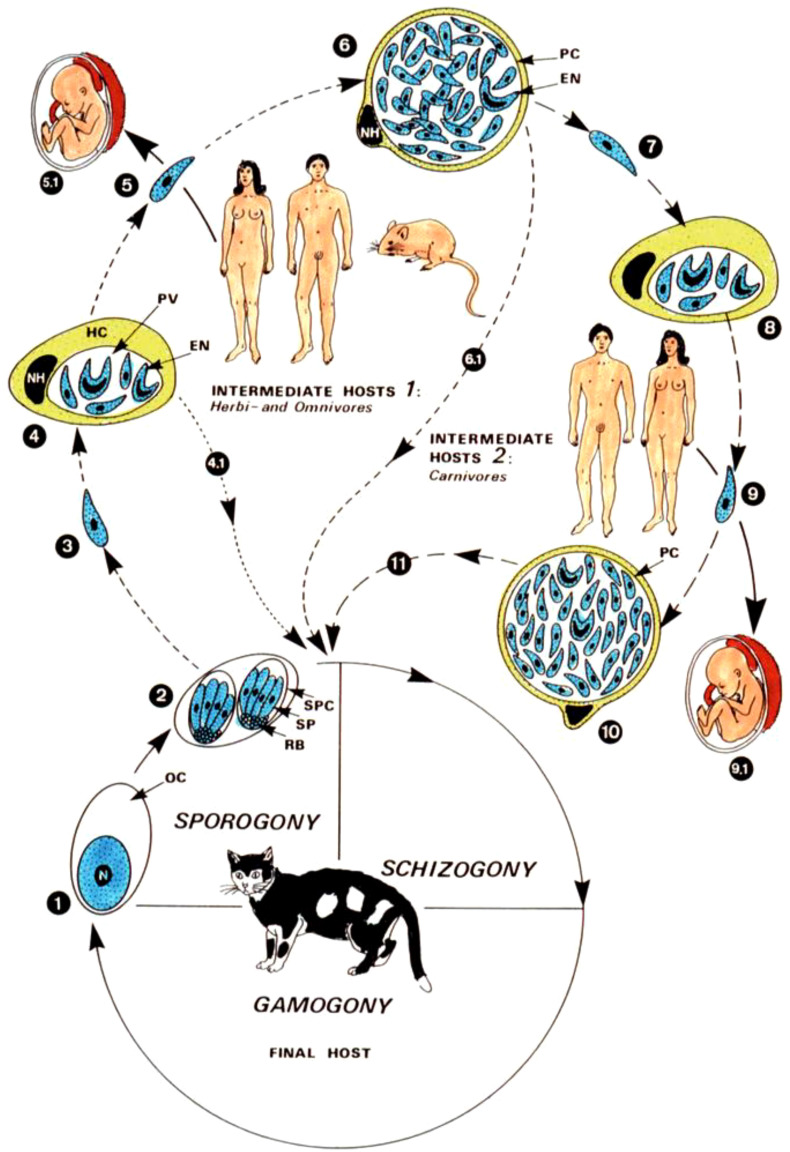
Development cycle and transmission routes of Toxoplasma gondii. The typical coccidial cycle takes place in the intestinal epithelium of felids, which become infected through oocysts (2), “pseudocysts” (8) and tissue cysts (6, 11). 1–11. Routes of infection in intermediate hosts. Source: Mehlhorn (28), Reproduced with permission from Springer Nature. © Springer-Verlag GmbH.

*T. gondii* is an intracellular parasite that not only causes infections, but can also influence the behavior of its hosts. One remarkable strategy of the parasite is to manipulate dopamine production in the brain. Studies have shown that *T. gondii* significantly increases dopamine release in infected nerve cells. It does this by expressing its own tyrosine hydroxylase, a key enzyme in dopamine synthesis, whereby the parasite directly increases dopamine production. This increase in dopamine levels has far-reaching effects on the host’s behavior. In rodents, *T. gondii* infection leads to a reduced fear of predators, especially cats, which increases their likelihood of being eaten - an advantage for the parasite’s life cycle (**Figure 1**). Similar behavioral changes have been observed in humans. Altered dopamine levels contribute to people’s increased risk-taking in everyday situations (23).

Another example of neurobiological influence by parasites is the modification of the immune response. Parasites such as *T. gondii* release substances that promote the secretion of anti-inflammatory molecules. These immunomodulatory effects can also affect the brain and alter neurotransmitter balance, which may be associated with changes in mood and behavior (11).

#### 2.3.2 Studies on personality changes caused by parasitic infections

Several studies have shown an association between *T. gondii* infections and personality changes in humans and other animals. This change in the fear response increases the likelihood that the rats will be eaten, which serves the life cycle of the parasite, as it requires its final host, the cat (3).

In humans, Flegr and other researchers have shown that infected individuals tend to exhibit behavioral patterns such as higher-risk behavior and lower impulse control. In a meta-analysis that took into account a large number of studies, changes such as increased risk-taking, neurotic behavior and slower reaction speed were found to be associated with *T. gondii* infection (8). Furthermore, some studies suggest that infected individuals are at higher risk of developing mental disorders such as schizophrenia and depression, possibly due to the neuroinflammatory response and dopaminergic dysregulation caused by *T. gondii* (24).

#### 2.3.3 Discussion of known cases and hypotheses

The mechanisms by which parasites influence behavior are intriguing and raise questions about evolutionary adaptation. It is speculated that parasites deliberately manipulate their hosts to increase their own chances of survival and dispersal. The “manipulation hypothesis” suggests that behavioral changes that make the host more susceptible to predation may help the parasite to return to its definitive host. The example of “fatal attraction” in infected rodents is a prominent example of this hypothesis (9). In addition to the aforementioned “manipulation hypothesis” of infected rodents, this hypothesis is also of interest in humans because behavioral changes could potentially have social and health implications. Although it is difficult to measure the direct influence of a parasite on complex human behaviors, numerous epidemiological studies support this hypothesis and show significant correlations between the prevalence of *T. gondii* and certain behavioral patterns and mental disorders (25, 26). Future research could further explore the exact molecular mechanisms by which parasites alter behavior and personality traits. Improved knowledge of the manipulation strategies could also contribute to the development of preventive and therapeutic measures to mitigate the possible negative effects of parasitic infections on behavior.

### 2.4 Sexual aggression as a behavioral effect

The ability of certain parasites to manipulate the behavior of their hosts potentially includes the enhancement of aggressive or sexual behavior. While research in this area is still in its early stages, there is evidence that parasitic infections can influence behavioral patterns that lead to aggressive and possibly also sexually aggressive behavior through mechanisms such as neurotransmitter changes and immune manipulation. *T. gondii* is a central focus of research here, as it’s known effects on the dopaminergic system and impulse control can influence behavioral tendencies such as risk-taking and aggression, which in turn could promote sexually aggressive acts (8). Dopamine and serotonin are two neurotransmitters strongly associated with the regulation of mood and social behaviors such as aggression and sexual arousal. By increasing dopamine production in infected cells, parasites such as *T. gondii* can enhance the drive for impulsive behavior, thereby lowering the threshold for aggressive impulses (18, 21). Some research has indicated that *T. gondii*-infected individuals tend to be more willing to take risks and have lower impulse control, which could lead to behaviors that are socially or sexually inappropriate (25). This increased willingness to take risks can make the individual more susceptible to situations that could lead to aggressive or violent acts, whereby sexually aggressive tendencies could also be intensified. In addition, certain social factors, such as stress or social isolation, may increase the likelihood of infected individuals displaying aggressive or sexually aggressive behavior (2). A higher sense of dominance and masculinity due to increased testosterone levels has been demonstrated in students (27). Another significant factor is the increase in dopamine levels caused by the parasite, which is considered a key mechanism for the intensification of aggressive and sexually aggressive behavior. Studies have shown that parasite-induced dopamine dysregulation can powerfully influence social interaction and attachment behavior, which could increase the potential for violent or aggressive impulses in social and sexual contexts (21).

In summary, the literature shows that parasitic infections can alter the behavior of their hosts through biochemical mechanisms such as influencing the neurotransmitter balance in the brain in a way that leads to an intensification of aggressive and potentially also sexually aggressive behaviors. Further research is needed to understand the exact mechanisms and extent of this influence and to develop possible preventive or therapeutic approaches to avoid such behavioral changes.

## 3 Discussion and implications for research

The results of research on behavioral changes caused by parasitic infections, especially those related to *T. gondii*, raise significant questions for psychology and medicine. These findings suggest that parasitic infections may pose more than just physical health risks, but may also have more profound effects on mental health and social behavior. The possibility that parasites influence human behavior – including aggressive or even sexually aggressive tendencies – calls for a re-evaluation of the role of infections in psychopathology and psychiatry (8, 26).

### 3.1 Implications for psychology and medicine

From a psychological perspective, the studies show that behavioral changes caused by *T. gondii* represent a form of subtle manipulation that goes beyond the traditional perspective of environmental and genetic factors. The fact that a biological agent can influence neurotransmitter regulation and behavioral patterns has far-reaching implications for our understanding of behavioral disorders and impulsivity. In medicine, this could lead to a re-evaluation of the treatment of behavioral disorders by considering potential infection as a factor, especially in the case of aggressive or impulsive behavior (21).

### 3.2 Prevention and treatment of parasitic infections

Prevention measures are crucial to reduce the potential negative behavioral effects of *T. gondii* infection. Public health initiatives could aim at increasing awareness of transmission routes, especially contact with cats or consumption of undercooked meat, as these are major routes of transmission for *T. gondii* (10). In addition, the development of medications or vaccines to prevent the infection would be an important step in mitigating the potential behavioral effects.

For those already infected, antipsychotic or anti-inflammatory therapies show promise for reducing behavioral effects. Studies indicate that medications that affect the dopaminergic system may offer a way to mitigate behavioral changes caused by *T. gondii*. However, the use of such therapeutic approaches is still experimental, and further clinical studies are needed to establish their efficacy and safety (2).

### 3.3 Implications for future research

The study of behavioral changes caused by parasitic infections opens up a new field of research that could broaden our understanding of mental disorders. Future studies could focus on further elucidating the specific mechanisms by which parasites affect human behavior, particularly by investigating neurotransmitter dysregulation and immune response. The role of inflammatory processes in the brain and their influence on behavior is of particular interest here and could lead to new therapeutic targets (11). In addition, longitudinal studies could help to better understand the long-term behavioral consequences of *T. gondii* infection, including potential risks for developing mental illness. Developing intervention studies that test preventive and therapeutic measures against the behavioral effects of parasite infections would be a further step towards minimizing the negative consequences and deepening our understanding of psychopathologies (24). Overall, these findings highlight the need to consider parasitic infections not only as a medical but also as a psychological problem. Further research could broaden our understanding of the interplay between infections and behavior and help develop prevention and treatment options for the complex behavioral effects of such infections.

## 4 Conclusion

The study of the effects of parasitic infections, in particular *T. gondii*, on human behavior shows that such infections can have profound and complex effects on the central nervous system. Analysis shows that *T. gondii* can cause behavioral changes through mechanisms such as manipulation of dopamine and serotonin levels, as well as inflammatory responses in the brain, leading to increased risk-taking and aggressive tendencies (18, 21). It is particularly noteworthy that these changes could potentially also play a role in social and sexual contexts. Studies in animals and humans indicate personality changes and an increased risk of mental disorders, which calls for a reorientation in research on mental and behavioral disorders (8, 24).

The significance of these findings for society is considerable. The possible behavioral changes caused by a widespread parasitic infection such as *T. gondii* underscore that such infections can pose not only medical but also psychological and social challenges. In particular, the potential for parasitic infections to exacerbate aggressive and impulsive behavior raises important questions for understanding and preventing violence and behavioral disorders. The research results suggest that broader education about transmission routes and risks, combined with preventive measures and innovative therapeutic approaches, could be a promising way to mitigate the negative effects of parasitic infections on behavior (2, 10).

This topic is highly relevant for future research, as the mechanisms by which parasites affect behavior are not yet fully understood. Further studies could examine the molecular and neurobiological processes in more detail to gain a deeper understanding of the interactions between parasite infections and human behavior. Furthermore, it would be useful to develop targeted intervention and prevention strategies and test them in controlled studies. Research into the long-term effects of such infections on mental health and social behavior in humans could provide important insights for the prevention and treatment of mental disorders and expand our understanding of the complex causes of human behavior (11, 25).

Overall, this work shows that parasitic infections can have more far-reaching effects than previously thought. The realization that parasites such as *T. gondii* may contribute to the development of aggressive and impulsive behavior patterns challenges both psychology and medicine to take infections seriously as a potential behavioral factor. This interdisciplinary research could make an important contribution to a better understanding of the underlying biological mechanisms and to the development of effective prevention and treatment approaches for the diverse effects of parasitic infections.
