# Correction: Sexually aggressive behavior triggered by parasitic infection – how parasites can influence our personality

**DOI:** 10.3389/fpsyt.2025.1639610

**Published:** 2025-07-16

**Authors:** Marco Goczol

**Affiliations:** ^1^ Department of Forensic Medicine, Medical Faculty, Leipzig University, Leipzig, Germany; ^2^ Department of National Security, Faculty of Information Sciences, University of Library Studies and Information Technologies, Sofia, Bulgaria

**Keywords:** personality change, parasitic infection, sexual aggression, *Toxoplasma gondii*, neuropsychiatric, psychological behavior

There was a mistake in the caption of **Figure 1** as published. The numbers in the caption were erroneously linked to the reference list instead of indicating points in the figure. Furthermore, the original source of the figure was not acknowledged. The corrected caption of **Figure 1** appears below:

**Figure 1:**
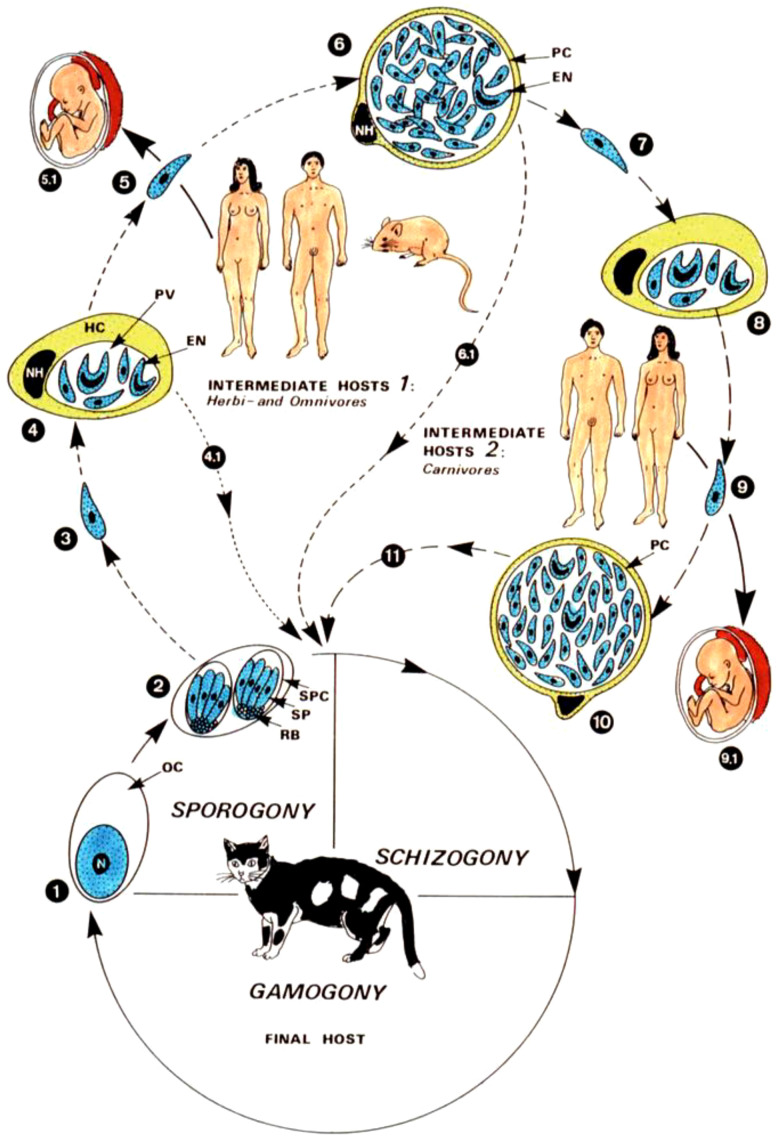
Development cycle and transmission routes of Toxoplasma gondii. The typical coccidial cycle takes place in the intestinal epithelium of felids, which become infected through oocysts (2), “pseudocysts” (8) and tissue cysts (6, 11). 1–11. Routes of infection in intermediate hosts. Source: Mehlhorn (28), Reproduced with permission from Springer Nature. © Springer-Verlag GmbH.

“Development cycle and transmission routes of Toxoplasma gondii. The typical coccidial cycle takes place in the intestinal epithelium of felids, which become infected through oocysts (2), “pseudocysts” (8) and tissue cysts (6, 11). 1–11. Routes of infection in intermediate hosts. Source: Mehlhorn (28), Reproduced with permission from Springer Nature. © Springer-Verlag GmbH”.

Refernece 8 was erroneously written as “Flegr J. How and why *toxoplasma gondii* influences human behavior. *Zoonoses Public Health*. (2013) 60:116–20. doi: 10.1016/j.pt.2013.01.007”. It should be “Flegr J. How and why Toxoplasma makes us crazy. Trends in parasitology. (2013) 29(4):156–63. doi: 10.1016/j.pt.2013.01.007”.

Reference 28 was erroneously written as “Mehlhorn H. Die Parasiten des Menschen. Erkrankungen erkennen, bekämpfen und vorbeugen. *Auflage*. (2022) 8:82”. It should be “Mehlhorn H. Die Parasiten des Menschen. Erkrankungen erkennen, bekämpfen und vorbeugen. 8th ed. Berlin, Heidelberg: Springer Spektrum. (2022). p. 82, doi:10.1007/978-3-662-65315-9”.

The original version of this article has been updated.

